# The Grain Growth Control of ZnO-V_2_O_5_ Based Varistors by PrMnO_3_ Addition

**DOI:** 10.3390/mi13020214

**Published:** 2022-01-29

**Authors:** Maofeng Xu, Changkun Cai, Yu Shi, Manyi Xie, Yanlong Wu, Yuanyuan Liu, Jun Peng, Jinxiao Bao, Shengli An

**Affiliations:** 1 School of Materials and Metallurgy, Inner Mongolia University of Science and Technology, Baotou 014010, China; montekane@163.com (M.X.); changkun_cai@126.com (C.C.); sharkv8088@163.com (Y.S.); manyixie0211@126.com (M.X.); wyl107307@163.com (Y.W.); liuyyk@163.com (Y.L.); pengjun@imust.edu.cn (J.P.); baojinxiao@imust.edu.cn (J.B.); 2 Inner Mongolia Key Laboratory of Advanced Ceramic Materials and Devices, Inner Mongolia University of Science and Technology, Baotou 014010, China; 3 Key Laboratory of Green Extraction & Efficient Utilization of Light Rare-Earth Resources, Ministry of Education, Baotou 014010, China

**Keywords:** abnormal grain growth, ZnO, V_2_O_5_, varistor

## Abstract

In this study, the grain growth behaviour of ZnO-V_2_O_5_-based ceramics with 0.25–0.75 mol% additions of PrMnO_3_ was systematically investigated during sintering from 850 °C to 925 °C. with the aim to control the ZnO grain size for their application as varistors. It was found that with the increased addition of PrMnO_3_, in addition to the decrease in the average grain size, the grain size distribution also narrowed and eventually changed from a bimodal to unimodal distribution after a 0.75 mol% PrMnO_3_ addition. The grain growth control was achieved by a pinning effect of the secondary ZnCr_2_O_4_ and PrVO_4_ phases at the ZnO grain boundaries. The apparent activation energy of the ZnO grain growth in these ceramics was found to increase with increased additions of PrVO_4_, hence the observed reduction in the ZnO grain sizes.

## 1. Introduction

ZnO-Bi_2_O_3_ ceramics are an established class of ZnO-based varistors. It is well known that the nonlinear current–voltage (*I*–*V*) characteristics of these ZnO-Bi_2_O_3_ varistors are directly dependent on their microstructures, mainly the average grain size and size distribution of ZnO [1]. Typically, a large average grain size of >30 μm is required for low-voltage applications, and a small average grain size of <10 μm is needed for high-voltage applications [2]. Additionally, a narrow grain size distribution is also critical for achieving stability of the electrical field strength of these materials [3].

It has been found that ZnO-V_2_O_5_ ceramics also exhibit a nonlinear *I*–*V* behaviour comparable to ZnO-Bi_2_O_3_ ceramics [4,5,6] and, thus, have the potential to be a new class of varistors [7]. One of the significant advantages of ZnO-V_2_O_5_ ceramics is that they can be sintered at a relatively low temperature of ~900 °C [8,9,10,11]. This outstanding feature allows these ceramics to be co-fired with Ag (m.p. 961 °C). This enables Ag, instead of expensive Pd or Pt, to be used as inner electrodes for applications in multilayer chip components [12]. Moreover, V_2_O_5_ is also a better sintering aid, compared with Bi_2_O_3_ for ZnO, enabling ZnO-V_2_O_5_ based ceramics to be densified to the same density at a lower temperature than ZnO-Bi_2_O_3_ based ceramics [13,14,15,16].

However, ZnO-V_2_O_5_ based ceramics suffer from a distinct disadvantage in that they have been shown to exhibit abnormal ZnO grain growth [17]. This is commonly attributed to the high reactivity of the V_2_O_5_-rich liquid phase formed during sintering. This phase assists in the diffusion of Zn^2+^ and thus promotes ZnO grain growth, which results in the formation of abnormally grown grains (AGG) of ZnO.

One approach to curb the formation of AGG in ZnO-V_2_O_5_ ceramics is to induce the formation of a secondary phase to hinder ZnO grain growth by the addition of a third oxide to the system [18,19]. The two commonly used additives are Cr_2_O_3_ and Sb_2_O_3_, as Cr_2_O_3_ can react with ZnO to form a ZnCr_2_O_4_ spinel phase, while Sb_2_O_3_ and ZnO form spinel phases of ZnSb_2_O_6_ and Zn_2.33_Sb_0.67_O_4_ [20,21,22]. However, these two additives have their own specific limitations. With Cr_2_O_3_, an addition greater than 1 mol% is required to effectively counter the AGG and refine the ZnO grain size in ZnO-V_2_O_5_ ceramics. However, at this required addition level, Cr_2_O_3_ has also been found to segregate at grain-boundary regions, leading to the deterioration of *I–V* behaviour due to high leakage current. With Sb_2_O_3_, the addition of up to 2 mol% is needed to control the grain growth of ZnO-V_2_O_5_; however, the addition of Sb_2_O_3_ increases the required sintering temperature for the system. At an Sb_2_O_3_ content greater than 0.5 mol%, the sintering temperature could increase to 1200 °C [23], nullifying the advantage of having the ZnO-V_2_O_5_-based system in the first place.

This study investigated the effect of increased additions of PrMnO_3_ on the grain growth of ZnO-V_2_O_5_ ceramics, with the aim to control the average grain size and size distribution of ZnO to improve their electrical properties as practical varistors.

## 2. Experimental

### 2.1. Sample Preparation

All reagents used in this research were of analytical grade (>99.5% purity) and were supplied by SINOPHARM. PrMnO_3_ powder was prepared in-house by mixing Pr_6_O_11_ and MnCO_3_ at a molar ratio of 1:6 and allowing the mixture to react at 1000 °C for 5 h in air. The formation of the PrMnO_3_ phase was confirmed by X-ray powder diffraction (XRD) analysis.

Table 1 summarises the nominal compositions of constituent powders used to prepare four ZnO-V_2_O_5_ ceramic samples and their designated sample names. The PrMnO_3_ powder was introduced with the aim to regulate the grain growth in these ceramics.

For each nominal composition, the powder mixture was homogenised by ball milling in absolute alcohol using zirconia balls in a polypropylene container on a planetary mill for 24 h. After milling, the powder mixture was dried at 80 °C for 24 h. The dried powder mixture was then mixed with a 5 wt% polyvinyl alcohol (PVA) binder and pressed into pellets of Φ12 mm × 1 mm under a uniaxial pressure of 130 MPa. The green pellets were first fired at 500 °C for 1 h to remove the binder before being subjected to different sintering temperature/time regimes in an alumina crucible at a heating rate of 4 °C/min. In this study, seven sintered ceramic discs for each nominal composition were produced at 850 °C, 900 °C, and 925 °C for 4 h and at 875 °C for 2, 4, 6, and 8 h. After sintering, the furnace was powered off to allow the sintered discs to cool down naturally within the furnace.

### 2.2. Sample Characterisation

The phase compositions of the sintered and quenched samples were analysed by XRD patterns obtained using a diffractometer (PANalytical X’pert Powder) with Cu K_α1_ radiation (λ = 0.1541 nm).

The microstructure of the sintered samples was observed and analysed by scanning electron microscopy (SEM) using a ZEISS Supra 55 electron microscope. A polished cross-sectional area along the thickness of the sintered disc was prepared and chemically etched in a dilute HCl solution to reveal the grain and phase structures for SEM observation.

The average ZnO grain size and grain size distribution were analysed using a representative SEM image of each sintered sample.

To determine the average grain size, the method reported by Mendelson [24] was used. Basically, the average grain-boundary intercept length ($\bar{L}$) was first determined by measuring along 10 randomly drawn lines across the SEM image. The average grain size *G* is then calculated by Equation (1) as follows:

(1) $$G=1.56\bar{L},$$

To determine the ZnO grain size distribution, the dimensions around 500–800 ZnO grains of each sample were measured from its SEM images using a dedicated image analysis software. The surface area (S) of each grain was then estimated, and the equivalent diameter (d) of each grain was obtained by transforming the surface area of the irregularly shaped grain into a circle of the same area using the method reported by Daneu [25].

The grain growth behaviour was analysed using a phenomenological kinetic grain growth equation established by Nicholson et al., which has been widely used for ZnO-based ceramic systems. The equation is expressed as follows:

(2) $$G^{n}-G_{0}^{n}=K_{0}t\exp\left(\frac{-Q}{RT}\right),$$

where *G* is the average grain size of the ZnO ceramic at time *t*, *G*_0_ is the initial grain size of the ZnO powder, *n* is the kinetic grain growth exponent, *Q* is the apparent activation energy, *R* is the universal gas constant, *T* is the absolute temperature, and *K*_0_ is a *T*-independent constant. In the case where *G*_0_ is significantly smaller than *G*, then Equation (2) is simplified to

(3) $$G^{n}=K_{0}t\exp\left(\frac{-Q}{RT}\right),$$

Equation (3) was used throughout this study to analyse the sintering behaviour of the ZnO-V_2_O_5_ samples studied in this study.

### 2.3. Sample I–V Characteristics

The DC current-voltage (I–V) behaviour of the sintered samples was measured using a withstand voltage tester (MS2671A, Xi’an Instruments Inc., Xi’an, China). The sintered discs (~Φ8 mm × 1 mm thickness) were painted with a silver paste on both surfaces to enable the measurements. The I–V  curve was determined by measuring the current at a stepwise-increased applied voltage until I reached 10 mA. The field strength (E) is defined as the applied voltage per sample thickness, and the current density (J) is the measured current per sample area. The switching field strength *E*_1mA/cm_^2^ is the field strength at *J* = 1 mA·cm^−2^. The nonlinear coefficient (*α*) is calculated by Equation (4) as

(4) $$\alpha =1/\left(\log E_{\left(10\mathrm{mA}/{\mathrm{cm}}^{2}\right)}-\log E_{\left(1\mathrm{mA}/{\mathrm{cm}}^{2}\right)}\right)$$

where $E_{10\;\mathrm{mA}\cdot {\mathrm{cm}}^{-2}}\;$ and $E_{1\;\mathrm{mA}\cdot {\mathrm{cm}}^{-2}}\;$ are the field strengths at 10Ma·cm^−2^ and 1mA·cm^−2^, respectively. The higher the value of α is, the better it is for a varistor.

## 3. Results and Discussion

### 3.1. Phase Compositions and Distributions in Sintered Samples

**Figure 1:**
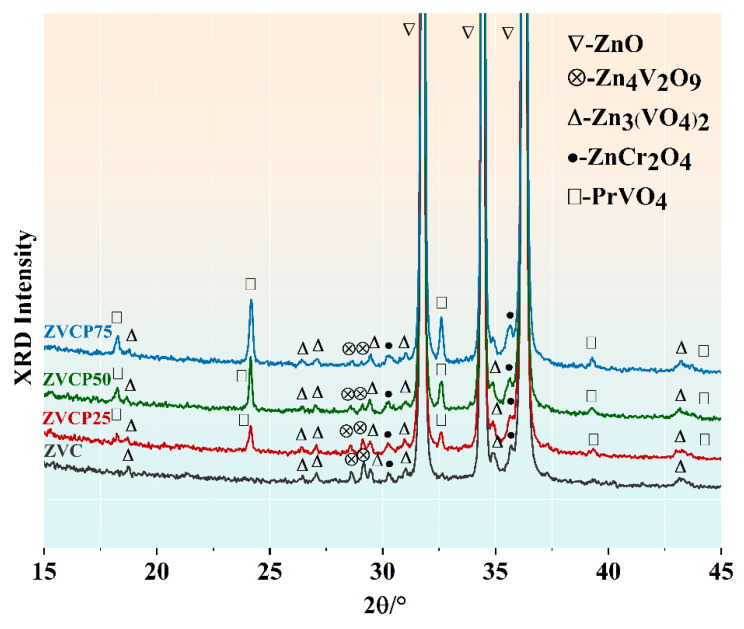
XRD patterns of the ZVC, ZVCP25, ZVCP50, and ZVCP75 samples sintered at 875 °C for 4 h.

Figure 1 shows the XRD patterns of the four ZVCP samples sintered at 875 °C for 4 h. For the ZVC sample, in addition to the main ZnO phase, several minor phases were also detected, including Zn_4_V_2_O_9_, α-Zn_3_(VO_4_)_2_, and ZnCr_2_O_4_. These minor phases have been commonly observed and reported in sintered ZnO-V_2_O_5_ systems. With the addition of PrMnO_3_, a new minor phase of PrVO_4_ was also observed in the ZVCP25, ZVCP50, and ZVCP75 samples. Consequently, this experimental result stated that the generation of PrVO_4_ could be attributed to the reaction between PrMnO_3_ and the V-contained phase. According to previous research studies, the formation of PrVO_4_ was reported as a product of phase transition between PrMnO_3_ and α-Zn_3_(VO_4_)_2_ with the following reaction:

(5) $$\alpha {\text{-}\mathrm{Zn}}_{3}{\left({\mathrm{VO}}_{4}\right)}_{2}+2\mathrm{Pr}{\mathrm{MnO}}_{3}\overset{}{\to }2\mathrm{Pr}{\mathrm{VO}}_{4}+{\mathrm{Mn}}_{2}O_{3}+3\mathrm{ZnO},$$

It should be noted that the mentioned phase transition did not produce only the PrVO_4_ phase. Additionally, the formation of the Mn_2_O_3_ phase was obtained in the same reaction. However, few lines of evidence indicating Mn_2_O_3_ in this phase observation can be found. As we know, Mn_2_O_3_ was helpful to generate the non-Ohm electrical property in ZnO varistor ceramic. Hence, the application of Mn_2_O_3_ was widely investigated by researchers. Related experimental results supported that Mn ion could easily be dissolved in ZnO grain. Thus, once the solid solution of ZnO-Mn_2_O_3_ formed, the diffraction peaks assigned to Mn_2_O_3_ vanished.

**Figure 2:**
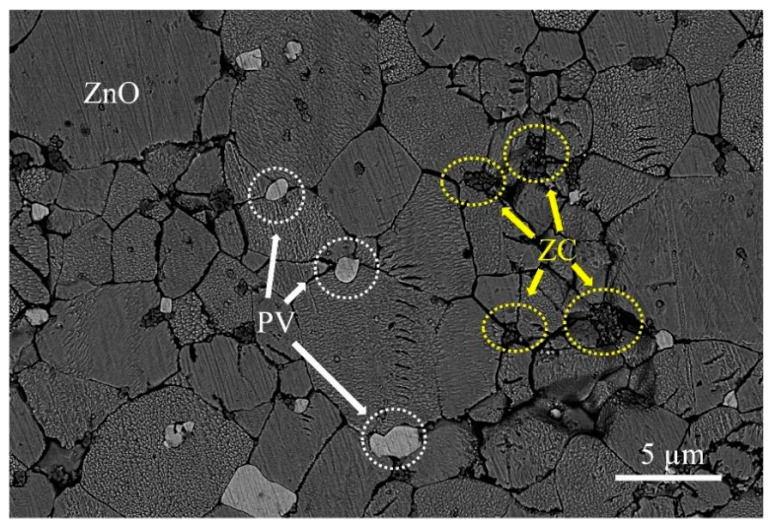
Back-scattered electron (BSE) image of a ZVCP50 sample sintered at 875 °C for 4 h.

**Figure 3:**
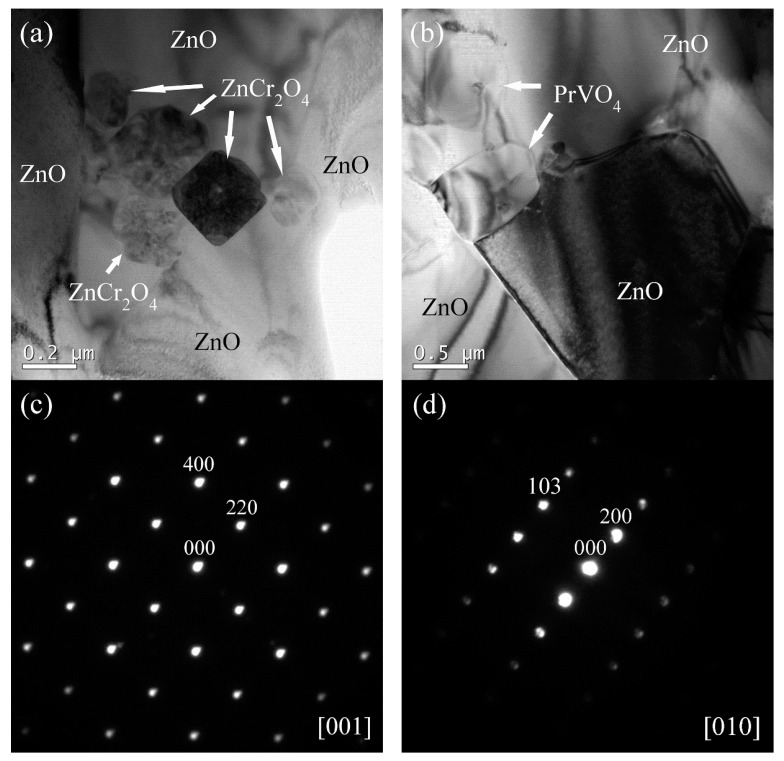
TEM micrographs and SAED patterns from secondary particle grains: (**a**) bright-field image of ZnCr_2_O_4_ grains from a sample with the composition of ZVCP75 sintered at 875 °C for 4 h, (**b**) bright-field image of PrVO_4_ grains from a sample with the composition of ZVCP75 sintered at 875 °C for 4 h, (**c**) electron diffraction pattern from the [001] zone of ZnCr_2_O_4_ grain in (**a**), and (**d**) electron diffraction pattern from the [010] zone of PrVO_4_ grain in (**b**).

Figure 2 shows a typical microstructure of a sintered ZVCP50 sample. As expected, the dominant morphology included grains of the ZnO phase, and the focus here was on the appearance and distribution of the minor phases. As seen in the micrograph, in addition to ZnCr_2_O_4_ (ZC) clusters (circled in yellow-dashed lines), PrVO_4_ (PV) grains (circled in white-dashed lines) were clearly seen to also distribute at the junctions of ZnO grains. In some cases, ZnO grains were observed to grow around either ZnCr_2_O_4_ clusters or PrVO_4_ grains. Figure 3 shows the TEM images, revealed the microstructural differences between PrVO_4_ and ZnCr_2_O_4_ phases. The electron diffraction pattern in Figure 3 further confirmed the existence of PrVO_4_ and ZnCr_2_O_4_. The ZnCr_2_O_4_ grains are very small—the grain size is below 0.2 µm—but the PrVO_4_ grains have a relatively larger size than ZnCr_2_O_4_, ranging from 0.5 µm to 4 µm. Most grains of ZnCr_2_O_4_ and PrVO_4_ were found located at the ZnO grain boundaries, and ZnCr_2_O_4_ grains tend to aggregate to form a heap of ~1 µm in size. This observation suggests that these secondary minor phases could exert a ‘pinning’ effect, which hindered the migration of ZnO grain boundaries and limited its grain growth.

### 3.2. Average ZnO Grain Sizes in Sintered Samples

For all four sintered samples, the average grain size of each sample under all sintering conditions was determined following the procedure described in Section 2.2, and the results are summarised in Table 2.

### 3.3. Kinetic Grain Growth Parameters

To reveal the effect of PrMnO_3_ addition on the sintering behaviour of the ZnO-V_2_O_5_ ceramics, the isothermal grain growth behaviour at 875 °C was first analysed using a rearranged Equation (3), as shown below:

(6) $$\log G=\frac{1}{n}\log t+\frac{1}{n}\left[\log K_{0}-0.434\left(\frac{Q}{RT}\right)\right]$$

**Figure 4:**
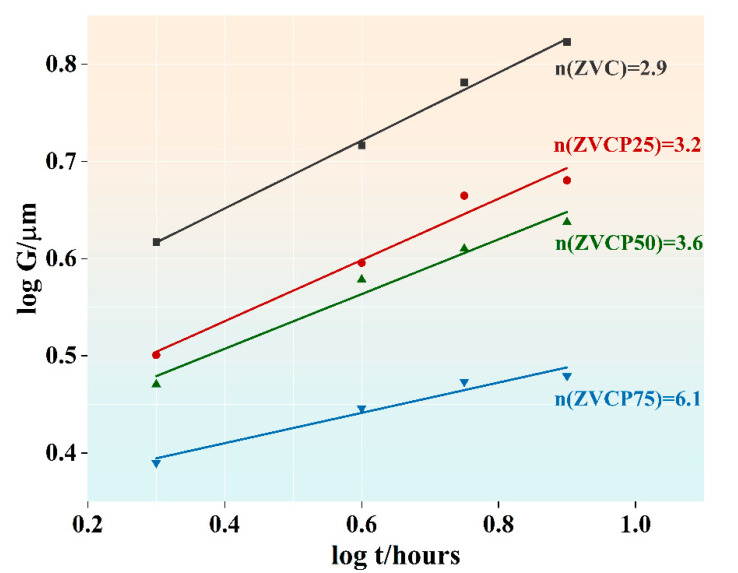
Grain growth behavior of the four samples sintered at 875 °C for 2–8 h.

Figure 4 illustrates logG vs. logt  curves, which show the isothermal grain growth behaviour of the four samples sintered at 875 °C for 2–8 h. A linear fit of the curves enabled the determination of the kinetic grain growth exponent n from the slope of the linear fit for each sample. The n values so determined are also shown in Figure 4. Notably, with the increased addition of PrMnO_3_, in addition to the decrease in the average grain size G, the grain growth rate also decreased, as indicated by the increased n  values.

To determine the apparent activation energy *Q* associated with the grain growth, Equation (3) is rearranged as

(7) $$\log\left(\frac{G^{n}}{t}\right)=\log K_{0}-\frac{0.434Q}{R}\left(\frac{1}{T}\right)$$

**Figure 5:**
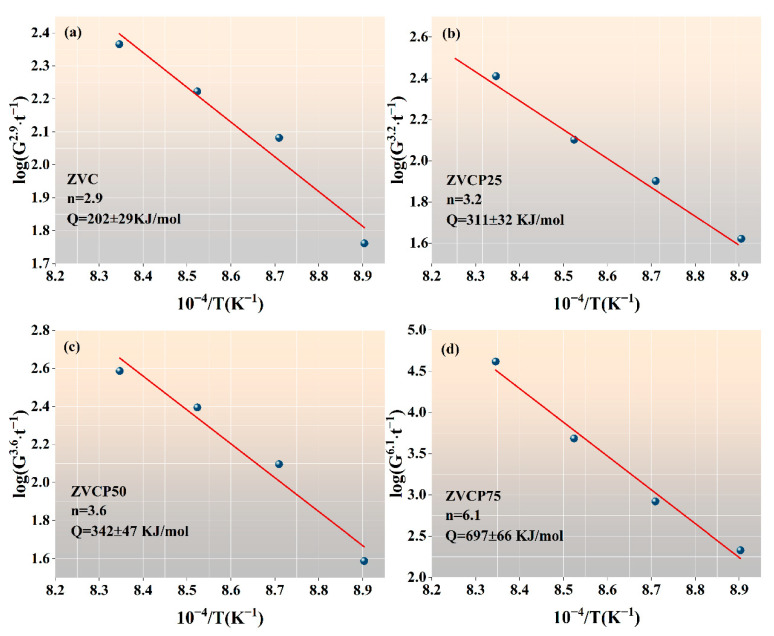
The log(*G^n^*/*t*) vs. (1/*T*) Arrhenius plots for grain growth of the four sintered samples: (**a**) ZVC, (**b**) ZVCP25, (**c**) ZVCP50, and (**d**) ZVCP75.

*Q* can then be calculated from the slope of the Arrhenius plot of log(*G^n^*/*t*) vs. (1/*T*). Figure 5 shows the Arrhenius plots of the four samples from the *G* values obtained at different sintering temperatures shown in Table 2. The apparent activation energy Q values determined from Figure 5 are summarised in Table 3, together with the n values determined from Figure 4.

As seen in Table 3, in both this study and other published studies, the basic ZnO-V_2_O_5_ binary systems had a kinetic grain growth exponent n  value spanning 1.5–1.8. This n value was lower than the n=3.0 observed for pure ZnO, indicating that the addition of V_2_O_5_ encouraged the grain growth of ZnO. This was attributed to the high reactivity of the V_2_O_5_-rich liquid phase during sintering, promoting the formation of abnormally grown ZnO grains.

The addition of a third component—e.g., the 0.35 mol% of Cr_2_O_3_ in this study or 0.5 mol% Sb_2_O_3_ as reported elsewhere—has been shown to increase the n value to 2.9 and 4.0, respectively. This means that the addition of a third component could hinder ZnO grain growth in basic ZnO-V_2_O_5_ binary systems. In both cases, this was attributed to the formation of spinel ZnCr_2_O_4_ or a ZnSb_2_O_4_ secondary phase, which exerts a pinning effect on ZnO grain boundaries and thus hinders ZnO grain growth.

It was clear that the addition of PrMnO_3_ to the ZnO + V_2_O_5_ (1 mol%) + Cr_2_O_3_ (0.35 mol%) ceramics resulted in a further increase in the *n* value, which increased to 6.1 after a nominal addition of 0.75 mol% of PrMnO_3_ for ZVCP75, demonstrating the high effectiveness of PrMnO_3_ in suppressing ZnO grain growth. As seen in the XRD and SEM analyses, the addition of PrMnO_3_ resulted in the formation of an additional spinel PrVO_4_ phase, which was also found to be distributed at the grain boundaries of ZnO similarly to ZnCr_2_O_4_ in the sintered ceramics. A similar pinning effect was thus believed to be the main reason for the hindered ZnO grain growth by the addition of PrMnO_3_.

As expected, it is clearly seen in Table 3 that the apparent activation energy values for the grain growth followed an inverse behaviour, compared with the n values. Generally, the higher the activation energy, the higher the n values, the slower the grain growth rate, and the smaller the average ZnO grain sizes.

### 3.4. Grain Size Distribution in Sintered Samples

**Figure 6:**
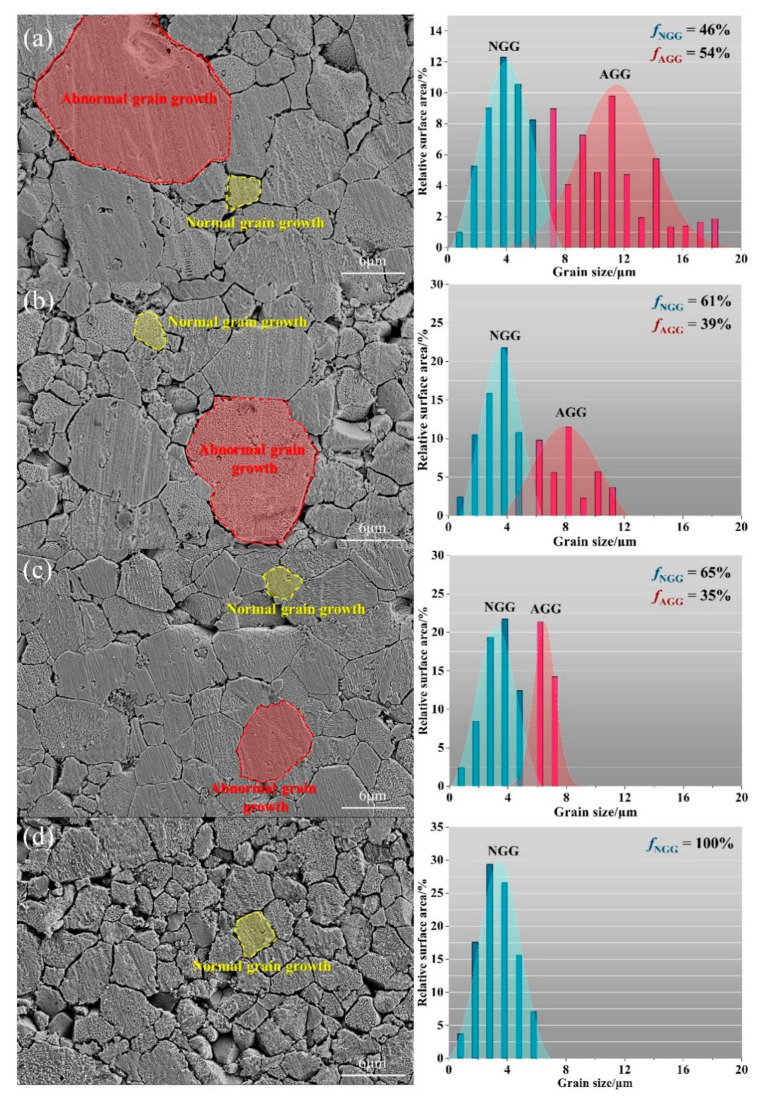
SEM microstructures and grain size distribution histograms of the samples sintered at 875 °C for 4 h: (**a**) ZVC, (**b**) ZVCP25, (**c**) ZVCP50, and (**d**) ZVCP75. NGG and $f_{NGG}$ refers to the normally grown grain and its number fraction. AGG and $f_{AGG}$ refer to the abnormally grown grain and its number fraction.

Figure 6 shows SEM images and grain size distribution histograms of the four samples sintered at 875 °C for 4 h. It is clear from the SEM images that with increased additions of PrMnO_3_, in addition to the decrease in the average grain size of ZnO, the size distribution also narrowed significantly.

It can be seen that the ZVC sample without the addition of PrMnO_3_ exhibited a broad and bimodal grain size distribution. In the context of this research, larger grains are considered to be the AGG, and the relatively smaller grains are considered as the normally grown grains (NGG). The area fractions of AGG and NGG for each sample are also included in the size distribution histograms in the figure. The addition of PrMnO_3_ resulted in the reduction and narrowing of the grain size range, in addition to diminishing the bimodal distribution. For ZVCP75, the grain size distribution became unimodal.

**Figure 7:**
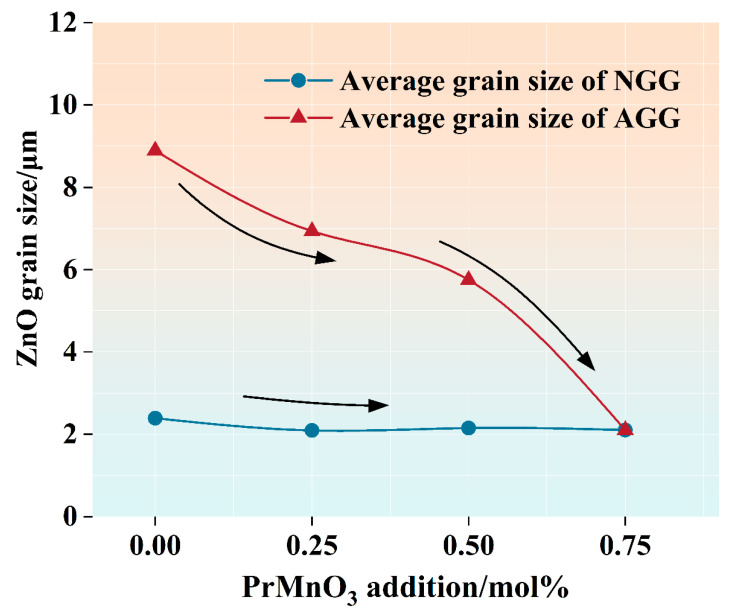
Effect of nominal addition of PrMnO_3_ on the average grain sizes of the AGG and NGG in ZVCP samples sintered at 875 °C for 4 h.

Figure 7 shows the average grain sizes of the AGG and NGG vs. the nominal addition of PrMnO_3_. This revealed the impact of PrMnO_3_ addition. Notably, the average grain size of NGG did not change much; however, the average grain size of the AGG decreased dramatically. This indicated the effectiveness of PrMnO_3_ addition in suppressing the coarsening of ZnO grains in this system. The number fractions of AGG ($f_{AGG}$) and NGG ($f_{NGG}$) were also determined for the sintered samples and are provided in the histograms.

### 3.5. Electrical Behaviour of Sintered Samples

**Figure 8:**
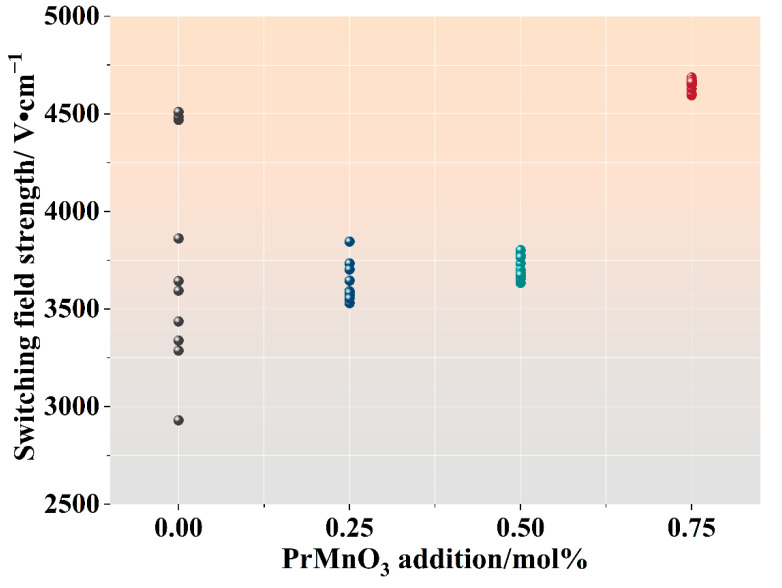
Measured switching field strength data vs. nominal addition amount of PrMnO_3_.

Figure 8 shows the switching field strength $E_{1\mathrm{mA}\cdot {\mathrm{cm}}^{-2}}$ of the four samples sintered at 875 °C for 4 h. For each sample, 10 specimens were prepared, their *I–V* curves were measured, and their $E_{1\mathrm{mA}\cdot {\mathrm{cm}}^{-2}}\;$ values were determined. Figure 8 shows all measured $E_{1\mathrm{mA}\cdot {\mathrm{cm}}^{-2}}\;$ data for the four samples. As seen, the basic ZVC sample displayed a very broad range of $E_{1\mathrm{mA}\cdot {\mathrm{cm}}^{-2}}$. Clearly, a 0.25 mol% nominal addition of PrMnO_3_ resulted in a remarkable narrowing of the $E_{1\mathrm{mA}\cdot {\mathrm{cm}}^{-2}}\;$ range for the ZVCP25 sample. Further increasing the PrMnO_3_ addition to 0.75 mol% produced a continuous narrowing of the $E_{1\mathrm{mA}\cdot {\mathrm{cm}}^{-2}}\;$ range.

This significant stabilisation of the switching field strength was attributed to the uniformisation of the ZnO grain size as the result of PrMnO_3_ addition. As seen in Figure 6, a 0.75 mol% addition of PrMnO_3_ in ZVC effectively eliminated the formation of abnormally grown ZnO grains. The resulting ZVCP75 sample was shown to have a markedly improved stability in its switching field strength. This was also consistent with the results reported by Hng et al. [12], which showed that the presence of just a few abnormally grown ZnO grains could have a pronounced destabilisation effect on the switching field strength of ZnO-V_2_O_5_ varistors.

Furthermore, with a 0.75 mol% addition of PrMnO_3_, there was also a marked increase in the $E_{1\;\mathrm{mA}\cdot {\mathrm{cm}}^{-2}}$ value for the ZVCP75 sample. This increase in switch field strength was mostly due to the refined ZnO grain size.

Table 4 summarises the relative density and number fraction of AGG and the average grain size of the sintered samples, together with the nonlinear coefficient and the average and range of the switching field strength of these samples.

It was noted that all the samples possessed a high sintering density at close to 95% of the theoretical density of ZnO. While the addition of PrMnO_3_ had little effect on the sintering density, it effectively reduced and eliminated the fraction of AGG and progressively refined the grain sizes of the resulting ceramics.

The electrical properties are attributed to the developed microstructures. The nonlinear coefficient also increased marginally from 7.2 to 8.9 from ZVC to ZVCP75, respectively, as seen in Table 4.

It was believed that a better sintering ability of ZnO ceramic can be achieved by adding V_2_O_5_ addition. During the sintering process, the generated α-Zn_3_(VO_4_)_2_ existed in the form of a liquid phase. Thus, the sintering mechanism was governed by solution–precipitation process. However, since the absolute homogeneous distribution of V_2_O_5_ in raw material was hardly achieved, regions that were rich in V_2_O_5_ produced more content of V-enriching liquid. In this case, the abnormal grain growth of ZnO begins in this liquid-phase-rich area, preferentially. To fix this problem, the traditional method needs the introduction of a secondary particle phase to hinder the migration of ZnO grain boundaries, such as the ZnCr_2_O_4_ spinel phase. However, a single ZnCr_2_O_4_ phase was insufficient to suppress all abnormally grown ZnO grains, as the distribution of the V-enriching liquid phase was still heterogeneous. Comparably, the PrVO_4_ particle phase plays a double role in the whole sintering process. Firstly, the PrVO_4_ particle hindered the migration of ZnO grain boundaries, similarly to ZnCr_2_O_4_. Additionally, the PrVO_4_ particle phase actually was the product of the reaction between PrMnO_3_ and α-Zn_3_(VO_4_)_2_. In a way, the generation of PrVO_4_ means the rearrangement of α-Zn_3_(VO_4_)_2_ in the ZnO matrix, which benefits the microstructural homogeneity. Moreover, the Mn_2_O_3_ generated by the mentioned reaction could optimise electrical properties. This was the reason why nonlinear coefficients were slightly enhanced by adding PrMnO_3_, even though α-Zn_3_(VO_4_)_2_ was sequentially consumed by added PrMnO_3_. In summary, we recognised that PrMnO_3_ addition can be treated as a potential additive in ZnO-V_2_O_5_-based varistor ceramic for its good performance on the repairmen of both grain growth control and electrical properties.

## 4. Conclusions

In this study, the effect of PrMnO_3_ addition (0.25 to 0.75 mol%) on the grain growth behaviour of ZnO-V_2_O_5_ (1 mol%)-Cr_2_O_3_ (0.35 mol%) ceramics was systematically investigated during sintering from 850 °C to 925 °C, with the aim to refine the ZnO grain structure. The main findings were as follows:

(1) The addition of PrMnO_3_ uniformised and refined the ZnO grains in the resulting ceramics. At a 0.75 mol% PrMnO_3_ addition, the bimodal grain size distribution was completely eliminated, and the average ZnO grain size was reduced by more than 45%, compared with the sample without PrMnO_3_ addition.
(2) The addition of PrMnO_3_ resulted in remarkably improved stability of the switching field strength, narrowing its range of variation from 1580 V/cm (without PrMnO_3_ addition) to only 91 V/cm (with 0.75 mol% PrMnO_3_ addition). The homogenisation and reduction in the ZnO grain sizes were responsible for the observed stabilisation of the switching field strength.
(3) A phenomenological analysis of the ZnO grain growth kinetics showed that the kinetic grain growth exponent n increased from 2.9 without PrMnO_3_ to 6.1 after a 0.75 mol% PrMnO_3_ addition, corresponding to an increase in apparent activation energy from 202 ± 29 to 697 ± 66 kJ/mol, respectively.
(4) The formation of a PrVO_4_ secondary phase as the result of PrMnO_3_ addition was responsible for the grain growth behaviour observed. The PrVO_4_ phase was found to be mostly located at the ZnO grain boundaries, thus hindering and eventually eliminating the abnormal growth of ZnO grains.

## Figures and Tables

**Table 1:** Basic compositions of the ZnO ceramics studied in this study.

Sample Name	Nominal Powder Compositions
ZVC	ZnO + V_2_O_5_ (1 mol%) + Cr_2_O_3_ (0.35 mol%)
ZVCP25	ZnO + V_2_O_5_ (1 mol%) + Cr_2_O_3_ (0.35 mol%) + PrMnO_3_ (0.25 mol%)
ZVCP50	ZnO + V_2_O_5_ (1 mol%) + Cr_2_O_3_ (0.35 mol%) + PrMnO_3_ (0.50 mol%)
ZVCP75	ZnO + V_2_O_5_ (1 mol%) + Cr_2_O_3_ (0.35 mol%) + PrMnO_3_ (0.75 mol%)

**Table 2:** Average grain sizes estimated from SEM analysis of ZVC and ZVCP samples sintered at different conditions.

Sample	Average Grain Size *G* (μm)						
	850 °C 4 h	900 °C 4 h	925 °C 4 h	875 °C 2 h	875 °C 4 h	875 °C 6 h	875 °C 8 h
ZVC	6.55	9.42	10.55	4.15	7.19	8.04	8.68
ZVCP25	4.95	7.14	9.65	3.18	5.94	6.62	6.80
ZVCP50	4.05	6.81	7.69	2.93	5.81	6.00	6.32
ZVCP75	3.02	5.04	6.76	2.47	5.80	4.95	5.14

**Table 3:** Summary of ZnO grain growth exponents and apparent activation energy for grain growth.

Ref	Material System (Nominal Composition, mol%)	Sintering Temperature *T* (°C)	Growth Exponent *n*	Apparent Activation Energy *Q* (kJ/mol)
[22]	Pure ZnO	900–1400	3.0	224 ± 16
[26]	ZnO-V_2_O_5_ (0.5–2.0)	900–1200	1.5–1.8	~88
[2]	ZnO-V_2_O_5_ (0.5–2.0)	900	1.2–1.6	–
[15]	ZnO-V_2_O_5_ (1.0)	750–1200	1.4	76 ± 7
[14]	ZnO-V_2_O_5_ (0.5) − Sb_2_O_3_ (0.5)	900–1050	4.0	365
This study	ZnO + V_2_O_5_ (1.0) + Cr_2_O_3_ (0.35)	875	2.9	202 ± 29
	ZnO + V_2_O_5_ (1.0) + Cr_2_O_3_ (0.35) + PrMnO_3_ (0.25)	875	3.2	311 ± 32
	ZnO + V_2_O_5_ (1.0) + Cr_2_O_3_ (0.35) + PrMnO_3_ (0.50)	875	3.6	342 ± 47
	ZnO + V_2_O_5_ (1.0) + Cr_2_O_3_ (0.35) + PrMnO_3_ (0.75)	875	6.1	697 ± 66

**Table 4:** Relative density, number fraction of AGG and average grain size, and electrical properties of the sintered samples at 875 °C for 4 h.

Sample	Relative Density (%)	$f_{AGG}$ (%)	Average Grain Size (µm)	Nonlinear Coefficient α	$E_{1\mathrm{mA}\cdot {\mathrm{cm}}^{-2}}\;(V/\mathrm{cm})$	
					Range	Average
ZVC	95.1	8.83	7.19	7.2	2924–4512	3649
ZVCP25	94.8	6.53	5.94	7.7	3523–3846	3632
ZVCP50	95.1	5.66	5.81	7.8	3629–3807	3726
ZVCP75	95.7	0	5.80	8.9	4594–4686	4620
